# Relevant mediators involved in and therapies targeting the inflammatory response induced by activation of the NLRP3 inflammasome in ischemic stroke

**DOI:** 10.1186/s12974-021-02137-8

**Published:** 2021-05-31

**Authors:** Qingxue Xu, Bo Zhao, Yingze Ye, Yina Li, Yonggang Zhang, Xiaoxing Xiong, Lijuan Gu

**Affiliations:** 1 Central Laboratory, Renmin Hospital of Wuhan University, Wuhan, 430060 China; 2 Department of Anesthesiology, Renmin Hospital of Wuhan University, Wuhan, 430060 China; 3 Department of Neurosurgery, Renmin Hospital of Wuhan University, Wuhan, 430060 China

**Keywords:** NLRP3 inflammasome, Ischemic stroke, Inflammation, Reactive oxygen species, Signaling pathway

## Abstract

The nucleotide-binding oligomerization domain (NOD)-like receptor (NLR) family pyrin domain-containing 3 (NLRP3) inflammasome is a member of the NLR family of inherent immune cell sensors. The NLRP3 inflammasome can detect tissue damage and pathogen invasion through innate immune cell sensor components commonly known as pattern recognition receptors (PRRs). PRRs promote activation of nuclear factor kappa B (NF-κB) pathways and the mitogen-activated protein kinase (MAPK) pathway, thus increasing the transcription of genes encoding proteins related to the NLRP3 inflammasome. The NLRP3 inflammasome is a complex with multiple components, including an NAIP, CIITA, HET-E, and TP1 (NACHT) domain; apoptosis-associated speck-like protein containing a CARD (ASC); and a leucine-rich repeat (LRR) domain. After ischemic stroke, the NLRP3 inflammasome can produce numerous proinflammatory cytokines, mediating nerve cell dysfunction and brain edema and ultimately leading to nerve cell death once activated. Ischemic stroke is a disease with high rates of mortality and disability worldwide and is being observed in increasingly younger populations. To date, there are no clearly effective therapeutic strategies for the clinical treatment of ischemic stroke. Understanding the NLRP3 inflammasome may provide novel ideas and approaches because targeting of upstream and downstream molecules in the NLRP3 pathway shows promise for ischemic stroke therapy. In this manuscript, we summarize the existing evidence regarding the composition and activation of the NLRP3 inflammasome, the molecules involved in inflammatory pathways, and corresponding drugs or molecules that exert effects after cerebral ischemia. This evidence may provide possible targets or new strategies for ischemic stroke therapy.

## Introduction

Stroke is a disease with high mortality and disability rates worldwide. Its prevalence rate increases annually, and the affected population is becoming increasingly younger. Stroke imparts heavy economic burdens on the families of patients and on society as a whole [1, 2]. According to its different etiologies and pathogeneses, stroke is clinically divided into hemorrhagic stroke, which is caused by cerebral vascular rupture, and ischemic stroke, which is triggered by arterial embolization or thromboembolism in the cerebrum. Ischemic stroke accounts for more than 80% of stroke cases, while hemorrhagic stroke accounts for fewer than 20% of cases. In this review, we focus on ischemic stroke because it is the condition with the second-highest mortality rate in the world after heart disease [3]. Focal ischemic stroke is the most common type of ischemic stroke [4–6]. There are many genetic and environmental risk factors for stroke. A study on single-gene diseases has suggested that common variants in approximately 35 sites are strongly related to the risk of stroke [7]. Additionally, various health-related and environmental factors, such as high blood pressure, diabetes, high cholesterol, high body mass index (BMI), smoking, and a history of stroke, all increase the risk of stroke [8]. The poststroke pathophysiological process is complicated and includes intracellular ion homeostasis disruption, acidosis, increased cytoplasmic Ca^2+^ concentrations, toxicity mediated by reactive oxygen species (ROS), bioenergy failure, arachidonic acid production, cytokine-mediated cytotoxicity, neuron and glial cells activation, complement activation, blood-brain barrier (BBB) destruction and leukocyte extravasation [9]. Above all, these factors can affect the functions and molecules of neurons, glial cells, and vascular cells [10]. Ischemic brain tissue injury is associated with the degree and duration of decreased blood flow. Greater severity and longer durations of obstruction are associated with more severe brain tissue injuries, including brain tissue edema, nerve cell dysfunction, and irreversible cerebral infarction, which lead to cognitive dysfunction and movement disorders [11]. Cerebral edema can also result in herniation and death [3]. When ischemic brain tissue is reperfused with blood, the cellular metabolic rate and oxygen consumption increase, leading to mitochondrial damage, increased ROS generation, increased cytoplasmic Ca^2+^ concentrations, and neutrophil infiltration and in turn aggravating cell damage via the inflammatory response [12]. This process is called cerebral ischemia/reperfusion (I/R) injury (IRI), which can be treated by targeting and attenuating inflammation through a series of therapeutic methods [13]. In addition, the ischemic penumbra has received much research attention. The penumbra is an area that can be saved to limit the negative effects of ischemic stroke [14], but there is a risk of the penumbra transforming into an ischemic zone, so interventions involving reperfusion of the penumbra after acute ischemic stroke warrant further consideration [15]. To date, there are no effective drugs for stroke treatment that do not elicit severe side effects. Intravenous tissue plasminogen activator (tPA) treatment and intravascular thrombectomy have been shown to be efficient for the treatment of acute ischemic stroke. However, tPA treatment is restricted to a narrow window, which limits its clinical application [16, 17]. In addition, treatment of stroke with tPA increases the risk of cerebral hemorrhage and induces neuronal excitotoxicity [18]. Studies in vitro and in vivo have demonstrated that the single-chain form of tPA (sc-tPA) selectively strengthens transduction of the N-methyl-D-aspartate receptor (NMDAR) signal and enhances NMDAR-mediated Ca^2+^ influx and neurotoxicity in cultured cortical neurons [19]. Neuroprotectors reduce infarct volume and inhibit neuronal death in cultures and stroke animal models but have failed in clinical trials because of their harmful effects and inefficiency [20, 21]. Molecular danger signals called damage-associated molecular patterns (DAMPs), such as high-mobility group box 1 (HMGB1), are released after multiple types of cell damage. In addition, pathogen-associated molecular patterns (PAMPs) derived from bacteria can activate the innate immune system through pattern recognition receptors (PRRs), which has recently been highlighted as an important inflammatory mechanism. The PRRs of neurons and astrocytes then activate the mitogen-activated protein kinase (MAPK) and nuclear factor kappa B (NF-κB) pathways [22, 23]. The NLRP3 inflammasome is a key intermediate, and treatments targeting upstream and downstream signaling pathways of NLRP3 may be novel strategies for stroke therapy [24]. In this review, we describe the composition and activation of the NLRP3 inflammasome in ischemic stroke and the molecules involved in associated inflammatory pathways. In addition, possible targets and new strategies related to the NLRP3 inflammasome for the therapy of ischemic stroke are described.

## The NLRP3 inflammasome

NLRP3, a classic nucleotide-binding oligomerization domain (NOD)-like receptor (NLR), is the most widely studied inflammatory complex and is believed to be closely associated with aseptic inflammation [25]. The NLRP3 inflammasome includes three protein subunits, the adaptor protein, apoptosis-associated speck-like protein containing a CARD (ASC), the receptor NLRP3, and the effector pro-caspase-1 [26], and participates in various infectious, inflammatory, and immune diseases [27]. The NLRP3 receptor comprises three domains, including a central NAIP, CIITA, HET-E, and TP1 (NACHT) domain; an N-terminal pyrin domain (PYD); and a C-terminal leucine-rich repeat (LRR) domain [28]. NLRs are members of the PRR family. NLRs are intracellular microbial receptors, and some NLRs can perceive danger signals, such as the presence of bacterial RNA and flagella, through both PAMP recognition and DAMP recognition [29]. Particles such as silicon dioxide, aluminum hydroxide, and amyloid fibers cause lysosomal membrane rupture, which helps microbial molecules enter the cytoplasm, thereby activating the NLRP3 inflammasome [30, 31]. The NACHT domain participates in the formation of the NLRP3 receptor and forms the central core of the inflammasome with the participation of adenosine triphosphate (ATP) after activation of oligomerization. The PYD is necessary for the binding of the NLR protein to the adaptor protein ASC [32, 33]. The inflammasome acts as a molecular platform to activate caspase-1 and then modulate the innate immune response [34]. Caspase-1 serves as an effector agent to cleave proteins and process pro-interleukin (IL)-1β and pro-IL-18 into the mature forms IL-1β and IL-18, respectively, and these mature cytokines are secreted into the extracellular space to perform their functions. ASC, which is visible as specks in apoptotic cells [35], mediates pro-caspase-1 self-hydrolysis and subsequent pro-IL-1β maturation but is not significantly involved in caspase-1-mediated neuronal death [33]. One study has indicated that caspase-12 can suppress the NLRP3 inflammasome by disturbing caspase-1 activation, which is associated with susceptibility to sepsis [36]. MCC950, an NLRP3 inhibitor, can reduce the expression of NLRP3 in ischemic brain tissue, and we have discovered that an NLRP3 inhibitor can reduce neuronal apoptosis, cerebral infarct size, and neurological dysfunction [37].

## Molecular and other mechanisms involved in activation of the NLRP3 inflammasome

**Fig. 1:**
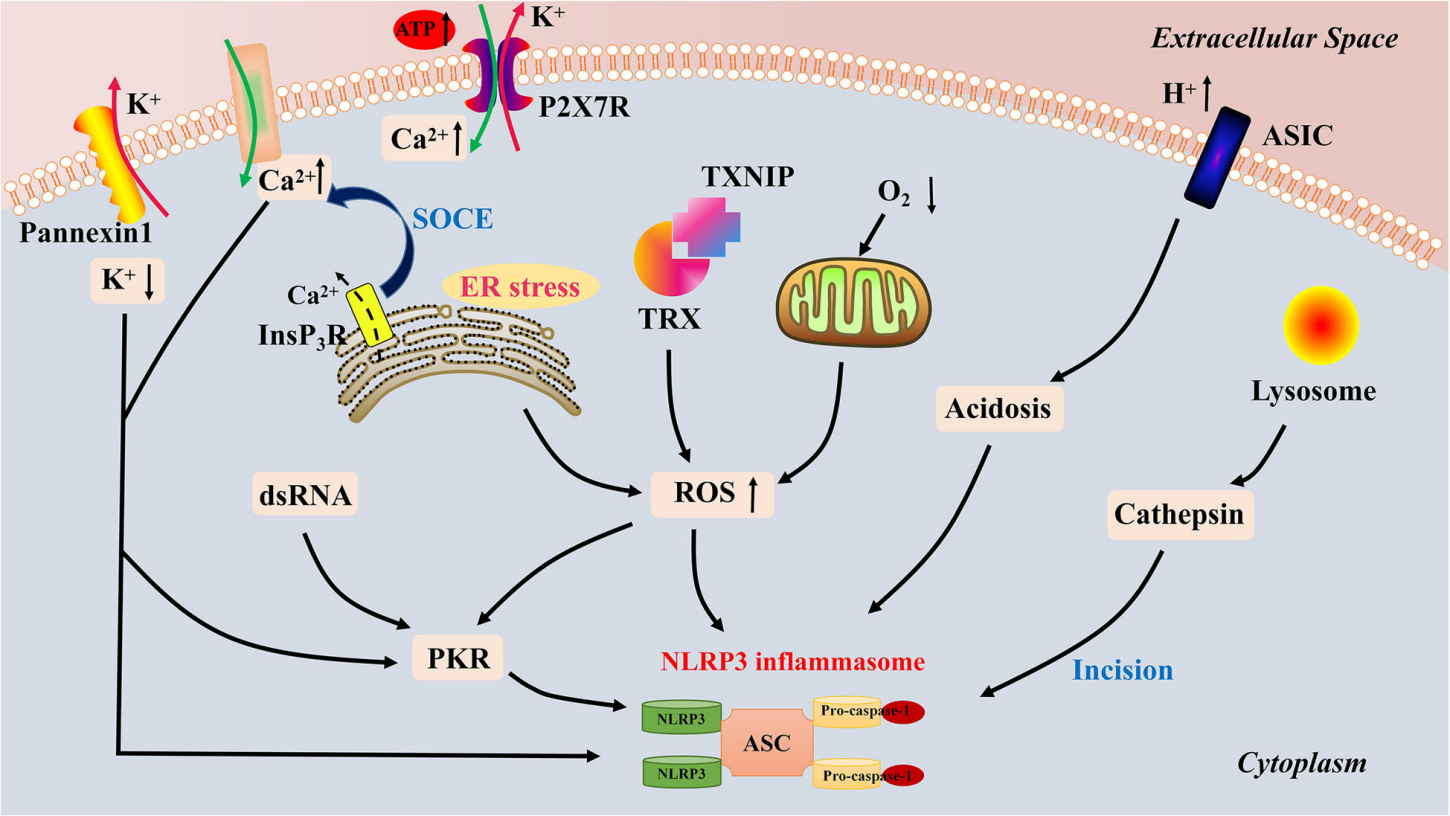
Mechanisms of NLRP3 inflammasome activation. Decreases in intracellular K^+^ concentrations, increases in intracellular Ca^2+^ concentrations, and excessive ROS production activate the NLRP3 inflammasome. As an inhibitor of the TRX system, TXNIP has been proven to mediate generation of large amounts of ROS and to activate the NLRP3 inflammasome. The activation of P2X7R caused by elevated ATP concentrations leads to increased intracellular Ca^2+^ concentrations and K^+^ outflow, resulting in activation of the NLRP3 inflammasome. Cathepsin is released into the cytoplasm after lysosomal membrane rupture, which induces activation of the NLRP3 inflammasome via cleavage of NLRP3 receptor-associated inhibitory domains or inhibitory proteins. dsRNA, increased intracellular Ca^2+^ levels, K^+^ outflow, increased ROS and other cellular stress factors activate PKR, and PKR activates the NLRP3 inflammasome. Anaerobic glycolysis results in the accumulation of large amounts of H^+^ and lactic acid, causing acidosis and ultimately activating the NLRP3 inflammasome

Activation of NLRP3 is thought to involve a variety of advanced signals, most of which are mutually synergistic but not exclusive. Studies have shown that NLRP3 receptors can sense disturbances in homeostasis and respond through several processes, as follows: (1) a low K^+^ concentration in the cytoplasm can trigger NLRP3 activation, (2) intracellular Ca^2+^ accumulation can induce harmful signaling pathways and activate the NLRP3 inflammasome, (3) lysosomal instability can stimulate cathepsin release and induce NLRP3 activation, and (4) mitochondrial injury-induced ROS production can activate NLRP3 and damage mitochondrial DNA (Fig. 1).

### K^+^-mediated activation of NLRP3

A decreased intracellular K^+^ concentration is a significant factor in NLRP3 inflammasome activation [38]. Studies have shown that reducing K^+^ levels in the cytoplasm induces NLRP3 inflammasome activation in vitro [39, 40]. Reductions in intracellular K^+^ levels can activate the NLRP3 inflammasome by influencing pore-forming or endogenous ion channels [41]. Notably, the K^+^ channel inhibitor glyburide efficiently inhibits inflammasome activation [42, 43]. Passive ATP release may be connected to P2X4 receptors in the plasma membranes of adjacent neurons and glial cells, closing receptors and resulting in K^+^ efflux [44, 45]. Damaged cells also passively release K^+^. Together, these mechanisms lead to increases in extracellular K^+^ concentrations and activation of membrane pannexin 1 channels [46]. Closure of pannexin 1 channels contributes to further ATP release and purinergic ligand-gated ion channel 7 receptor (P2X7R) activation, which restimulate the pannexin 1 channels to form a positive feedback loop, thus causing excessive K^+^ outflow and reducing inflow [44, 45, 47].

### Ca^2+^-mediated activation of NLRP3

Some studies have indicated that an increasing intracellular Ca^2+^ concentration activates the NLRP3 receptor both in vitro and in vivo [48]. During focal brain ischemia, high intracellular Ca^2+^ levels may be caused mainly by increased intracellular Ca^2+^ influx and decreased Ca^2+^ efflux due to increased intracellular Ca^2+^ release in damaged neurons and glial cells [49, 50]. Ca^2+^ release by injured cells can lead to intracellular Ca^2+^ overload through a specific mechanism. Briefly, necrotic cells in the ischemic core passively release Ca^2+^, increasing the extracellular Ca^2+^ concentration. The Ca^2+^ binds to and activates Ca^2+^-sensing receptors (CaSRs) and GPR6CA molecules on adjacent neuronal cells and glial cells, indirectly mediating NLRP3 receptor activation and simultaneously reducing intracellular cyclic adenosine monophosphate (cAMP) concentrations [51, 52]. Reductions in cytoplasmic cAMP concentrations can promote activation of the NLRP3 receptor [53–55]. After activation of cAMP and NLRP3, membrane-bound phospholipase C (PLC), which cleaves phosphatidylinositol-4,5-diphosphate (PIP2) into inositol triphosphate (InsP3) and diacylglycerol (DAG), can be activated [56, 57]. InsP3 interacts with the InsP3 receptor (InsP3-R) located in the endoplasmic reticulum (ER) to stimulate Ca^2+^ release [48, 52, 56, 57]. Experiments have shown that both ER Ca^2+^ release and extracellular Ca^2+^ influx are necessary for activation of the NLRP3 inflammasome. Pretreatment or incubation in Ca^2+^-free medium with brief exposure to thapsigargin (Tg), a suppressant of the sarcoplasmic/ER Ca^2+^-ATPase (SERCA) pump, attenuates activation of the NLRP3 inflammasome. ER-released Ca^2+^ can trigger extracellular Ca^2+^ influx through store-operated Ca^2+^ entry (SOCE) [58]. In addition, the increased concentration of Ca^2+^ leads to Ca^2+^ accumulation in the mitochondrial matrix through the mitochondrial Ca^2+^ uniporter (MCU) and the loss of mitochondrial transmembrane potential, thus activating the NLRP3 inflammasome. Other studies have shown that after the membrane attack complex (MAC) is formed, some Ca^2+^ is released through the ER via ryanodine receptors [59]. In conclusion, Ca^2+^ can indirectly activate the NLRP3 inflammasome through functional changes in the ER and mitochondria. The low-affinity Na^+^/Ca^2+^ exchanger (NCX) and high-affinity Ca^2+^-ATPase in the plasma membrane are the major transporters responsible for regulating intracellular Ca^2+^ levels and returning plasma Ca^2+^ concentrations to resting levels to maintain homeostasis [60]. Under conditions of excessive intracellular Na^+^, NCX activates its reverse exchange mode, mediating Ca^2+^ influx and Na^+^ efflux, which results in intracellular Ca^2+^ overload [61]. Selective inhibitors of the NCX reverse mode have been developed and tested in animal models of stroke and have been conclusively shown to reduce brain damage [62, 63].

### ROS-mediated activation of NLRP3

ROS play important roles in inflammation, oxidative stress, changes in blood vessel tension, and oxidation of low-density lipoprotein cholesterol (LDL-C) [64, 65]. ROS are also necessary for NLRP3 inflammasome activation. When the brain undergoes ischemic and hypoxic damage, large amounts of ROS are generated due to mitochondrial damage or insufficient oxygen supply, and these ROS in turn further disrupt mitochondrial function and structure [66]. There is evidence that mitochondria may be important sources of ROS related to NLRP3 inflammasome activation [67]. As byproducts of oxidative phosphorylation, ROS can be produced by mitochondria; these ROS not only continuously activate the NLRP3 inflammasome but also continue to damage mitochondria. When the ER is under stress, nicotinamide adenine dinucleotide phosphate (NADPH) oxidase (NOX) in the ER can induce ROS generation to restore ER homeostasis [68]. Excessive ROS production in the ER leads to mitochondrial Ca^2+^ deposition and further aggravates mitochondrial damage [69]. Mitochondrial autophagy is a crucial adjuster of NLRP3 activation because it eliminates dysfunctional and damaged mitochondria, thus ultimately reducing ROS levels. Increases in the numbers of damaged and dysfunctional mitochondria after treatment with mitochondrial autophagy inhibitors can enhance activation of the NLRP3 inflammasome [70]. Oxidized mitochondrial DNA can also lead to excitation of the NLRP3 receptor, consistent with the role of mitochondria in the signal transduction of the NLRP3 inflammasome [71]. During cerebral ischemia, ROS levels can increase through disturbance of the electron transport chain or activation of NOX, xanthine dehydrogenase, phospholipase A2 (PLA2), or nitric oxide synthase (NOS) [72–74]. A variety of experiments involving interference with mitochondrial function and uncoupling of the electron transport chain have proven that mitochondrial dysfunction can enhance ROS production and ultimately activate the NLRP3 receptor; this situation may occur after cerebral ischemia. When mitochondrial autophagy is disrupted, ROS production increases significantly, and the NLRP3 receptor is activated [75]. Some studies have shown that nuclear factor erythrocyte 2–related factor 2 (NRF2) is also necessary for NLRP3 activation [76, 77]. However, other studies have shown that NRF2 regulates antioxidant gene levels to support cell survival during oxidative stress while also inhibiting NLRP3 activity by limiting ROS levels [78].

### TXNIP-mediated activation of NLRP3

Thioredoxin (TRX)-interacting protein (TXNIP) is an endogenous suppressor of the TRX system that mainly inhibits cellular mercaptan production and has antioxidant and anti-inflammatory effects. However, increasing evidence has shown that TXNIP exerts its proinflammatory effect by activating the NLRP3 inflammasome [79]. In addition, numerous studies have shown that TXNIP may act as a primary mediator linking various undesirable stimuli, such as oxidative stress [80] and inflammation [81], to the NLRP3 inflammasome. TXNIP is a significant molecular site and signaling molecule in ER stress (ERS) and the inflammatory response [82]. In the context of ERS, TXNIP produces large amounts of ROS, which activate the NLRP3 inflammasome, resulting in IL-1β secretion [82, 83]. After ischemic stroke, TXNIP exacerbates cerebral injury through redox imbalance and subsequently activates the NLRP3 inflammasome [84]. In unaffected cells, TXNIP binds to and inhibits redox-related TRX, and the presence of the complex is associated with elevations in cytoplasmic ROS levels, which cause TXNIP to bind to NLRP3 receptor domains (mainly the LRR domain) and thus induce activation of NLRP3 receptors [5, 67, 85]. Recent evidence also suggests that NRF2, which is regarded as a pivotal molecule in the antioxidant stress system, inhibits TXNIP and NLRP3 inflammasome activation in IRI of the cerebrum [86]. This suggests that TXNIP-mediated activation of the NLRP3 inflammasome is a key factor in ischemic stroke. Therefore, the associated signaling pathway could be a new target for the treatment of ischemic stroke.

### P2X7R-mediated activation of NLRP3

P2X7R is a nonselective ATP-gated cation channel located on the membranes of various immune cells. Activation of P2X7R produces a range of harmful effects, including intracellular Ca^2+^ concentration increases, glutamate release, and NLRP3 inflammasome activation [37, 87]. These adverse effects may lead to aggravation of ischemic brain tissue injury and excitotoxicity and even to irreversible damage. Brilliant Blue G (BBG), an antagonist of P2X7R, can significantly reduce neuronal apoptosis, cerebral infarct size, and nerve function defects and save brain tissue. After ischemic stroke, ATP accumulates in damaged and inflamed tissue, and the increase in ATP concentration activates P2X7R, leading to intracellular K^+^ efflux that activates the NLRP3 inflammasome [88]. Therefore, the NLRP3 inflammasome has been reported to be a potential downstream signaling factor of P2X7R [89]. Thus, blocking P2X7R activation is a novel strategy for neuroprotection in the context of ischemic stroke.

### Cathepsin-mediated activation of NLRP3

The lysosomal cysteine protease cathepsin, a sterile particle, is released into the cytoplasm following lysosomal membrane destruction and rupture caused by stimulation with DAMP particulate crystals [90–92]. The released cathepsin induces NLRP3 inflammasome activation by cleaving the inhibitory domain or repressive protein associated with the NLRP3 receptor [93–95]. Sterile particles (including crystals and other factors originating from cholesterol clefts released by cerebral atherosclerotic vessels) as well as uric acid and Ca^2+^ released from crystals formed by necrotic tissues in the ischemic area can be absorbed into the lysosomes of various types of cells (including recruited astrocytes, microglia and infiltrating white blood cells) via engulfment, phagocytosis or scavenger receptor-mediated membrane absorption, which subsequently causes lysosomal instability [96–99]. In addition, some exogenous particulates, such as silica, alum, and asbestos, do not dissolve but are instead repeatedly phagocytosed and transported into lysosomes, which can cause lysosomal rupture and continuous cathepsin release [31]. The cathepsin lysosomal cysteine protease family is a family of 11 enzymes that were initially thought to function only in lysosomes; however, recent studies have shown that cathepsins have additional lysosomal and extracellular functions [100–102]. Cathepsin-B plays a crucial role in NLRP3 inflammasome activation, and a cathepsin-B inhibitor has been shown to specifically inhibit this process [103]. In addition, studies using combinations of multiple cathepsin inhibitors have indicated that multiple cathepsins can effectively activate the NLRP3 inflammasome [91, 104, 105]. Overall, the evidence indicates that cathepsins play key roles in NLRP3 activation, which provides new ideas for stroke treatment.

### PKR-mediated activation of NLRP3

Protein kinase R (PKR) belongs to the serine/threonine-protein kinase family, which is commonly activated by double-stranded RNA (dsRNA) [106, 107]. During cerebral ischemia, PKR is activated by various cellular stress factors, such as increased intracellular Ca^2+^ levels, K^+^ efflux, and elevated ROS levels; activated PKR then participates in the inflammatory response. PKR is an indispensable component for inflammasome assembly and activation [108]. A recent experiment demonstrated that complement 5a receptor 2 (C5aR2) promotes NLRP3 activation by increasing dsRNA-dependent PKR expression [109, 110]. We demonstrated that Epac1 regulates PKR phosphorylation, leading to inactivation, in retinal lysates generated from retinal endothelial cells (RECs) with Epac1-specific knockout. PKR inhibition experiments have demonstrated that PKR deficiency reduces the expression levels of NLRP3, caspase-1, and IL-1β. In addition, some studies have indicated that PKR modulates the NLRP3 inflammasome in RECs. Epac1 regulates PKR phosphorylation, leading to PKR inactivation and significantly reducing NLRP3 signaling [111]. PKR has also been reported to mediate harmful palmitic acid (PA)-induced inflammation through the Jun N-terminal kinase (JNK)/NF-κB/NLRP3 signaling pathway in cultured cardiomyocytes [112]. P58 can inhibit PKR and is associated with activation of inflammasomes. A comparison of primary bone marrow–derived macrophages (BMDMs) between p58-knockout mice and wild-type mice showed that p58 deficiency increases PKR and NF-κB activation and increases proinflammatory gene expression [113]. However, studies have also shown that activation of PKR is not required in ASC oligomers or the NLRP3 inflammasome when macrophages exposed to nonactivated anthrax lethal toxin undergo pyroptosis [114]. Moreover, in PKR-deficient macrophages, pro-IL-18 is still transformed into IL-18, indicating that PKR is not necessary in this context [115]. The above findings demonstrate that the roles of PKR in NLRP3 inflammasome activation and the inflammatory response still need to be clarified in future studies.

### Acidosis-mediated activation of NLRP3

After stroke, anaerobic glycolysis occurs due to vascular obstruction, cerebral tissue ischemia, and hypoxia. This process provides compensatory and transient energy support but produces large amounts of lactic acid and H^+^, leading to local pH decreases that cause irreversible cerebral cell death [116]. Lactic acidosis is thus strongly associated with ischemic stroke [117]. Acidosis is also regarded as an inducer of NLRP3 inflammasome activity. The NLRP3 receptor is activated by intracellular acidosis and reductions in intracellular K^+^ concentrations, although the mechanism remains to be clarified [118]. An acidic extracellular environment can trigger cell signaling events through changes in the surface or intracellular environment. On the cell surface, acid-sensitive ion channels (ASICs) directly activated by protons are triggered by acidosis to induce cell reactions [119]. ASICs are widely expressed in the central nervous system (CNS), including in the cerebral cortex, hippocampus, and cerebellum [120]. Ischemic cerebral acidosis leads to neuronal damage, and the damaging effect is mediated partly by ASICs [121, 122]. In cerebral ischemia, the passive release of H^+^ from necrotic cells results in extracellular acidosis in the ischemic area [118, 123]. Components of the acidic extracellular environment can bind to neurons and glial cells via H^+^-activated ASICs [124]. In the CNS, ASIC1 is the most widely distributed member of the ASIC family; in particular, the ASIC1a isomer is Ca^2+^ permeable and is associated with neuronal injury mediated by acidosis under ischemic conditions [121, 122, 125]. The use of ASIC blockers significantly reduces brain infarct volume, prevents damage, and has protective effects on the ischemic brain [121]. In addition, ASIC1 does not depend on the conduction of Ca^2+^, and ASIC1a-induced phosphorylation of receptor-interacting protein 1 (RIP1) has been found to be a new type of injury that occurs through the interaction of ASIC1a with the serine/threonine kinase RIP1 and subsequent induction of pyroptosis to mediate acidosis and ischemic cell death [126].

## Role of the NLRP3 inflammasome in ischemic stroke

### NLRP3 inflammasome–related neuronal death in ischemic stroke

**Fig. 2:**
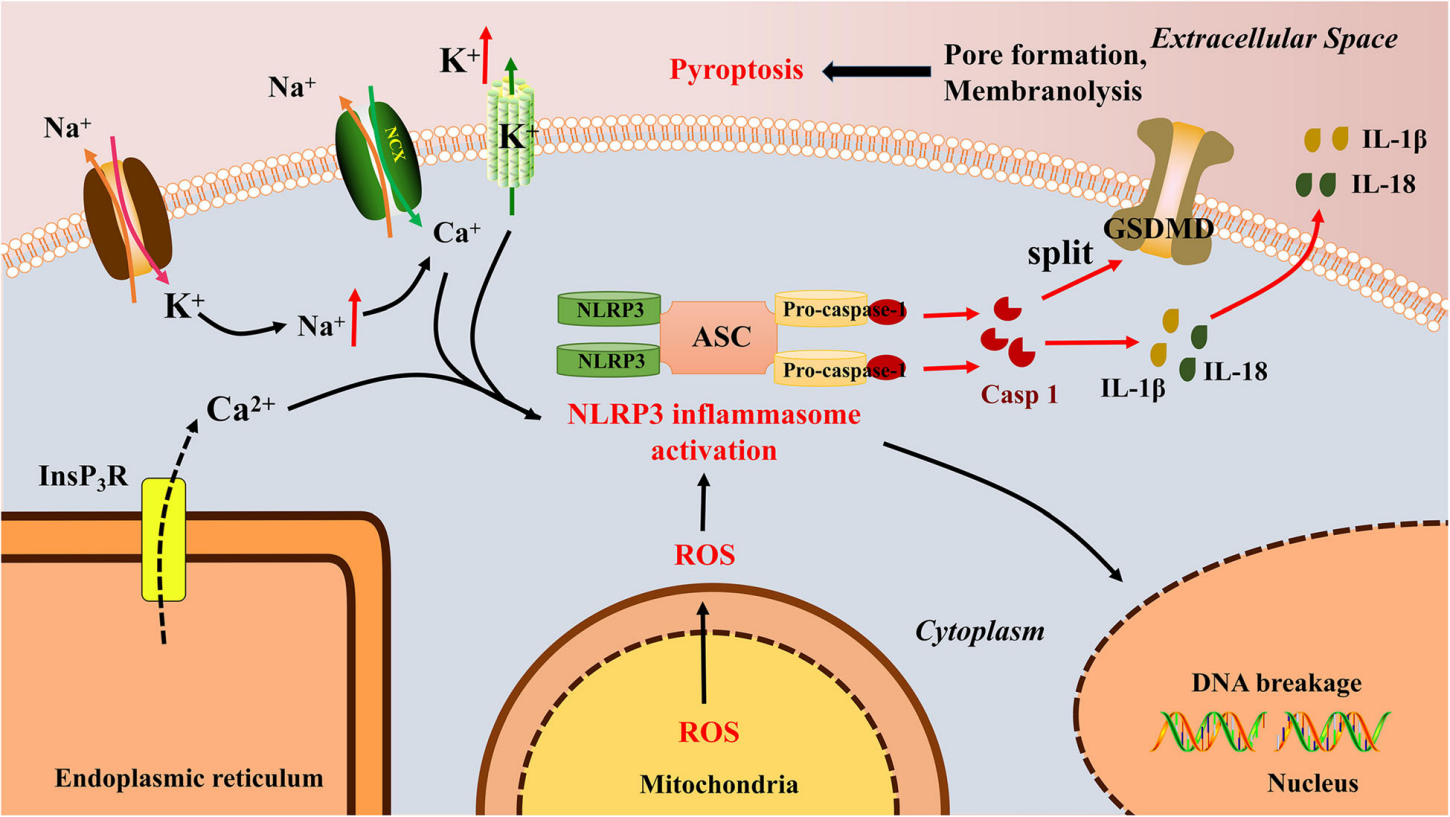
Metabolic changes in the intracellular and extracellular environments activate the NLRP3 inflammasome, leading to pyroptosis. Pyroptosis is characterized by GSDMD-mediated cell death. Extracellular and intracellular environments undergo metabolic changes, including reductions in ATP, efflux of intracellular K^+^, increases in intracellular Ca^2+^, and production of large amounts of ROS by mitochondria; the ROS cannot be normally removed, and the NLRP3 inflammasome is activated, prompting pro-caspase-1 to self-cleave into caspase-1. Then, caspase-1 lyses and activates GSDMD, leading to pore formation, membrane lysis, and DNA breakage.

Pyroptosis is a kind of programmed cell death that differs from necrosis and apoptosis and is induced entirely by cleaved caspase-1 to cause inflammatory cell death [127]. Caspase-1 dependence is characteristic of pyroptosis. Necrotic morphological changes, including membrane rupture, pore formation, and edema, promote the release of materials within inflammatory cells and damage to the actin cytoskeleton. In contrast, pyroptosis results in complete mitochondrial destruction and ballooning (small bubble formation) without the release of cytochrome-c (Cyt) [128–131]. At the molecular level, pyroptosis is characterized by gasdermin D (GSDMD)-mediated cell death [132]. Activated caspase-1 cleaves GSDMD and triggers intracellular GSDMD-N domain oligomerization, which results in the formation of pores to release IL-1β, IL-18, and HMGB1. In addition, active caspase-1 mediates pro-IL-1β/IL-18 maturation into IL-1β/IL-18, leading to inflammation [129, 133]. Caspase-1 thus cleaves and activates GSDMD, IL-1β, and IL-18, which then escape into the extracellular space through pores generated by GSDMD [132] (Fig. 2). Hence, GSDMD may be a potential therapeutic target to inhibit caspase-1-mediated pyroptosis [134]. After stroke, extracellular and intracellular environments undergo metabolic changes, including reductions in ATP, efflux of intracellular K^+^, increases in intracellular Ca^2+^, high mitochondrial production of ROS that cannot be eliminated via normal methods, and leukocyte recruitment to damaged sites. In the contexts of these stresses, the NLRP3 inflammasome is activated, prompting pro-caspase-1 self-cleavage for maturation. Caspase-1 then cleaves pro-IL-1β and pro-IL-18 into IL-1β and IL-18, respectively. In addition, during stroke, necrotic cells secrete danger signals called DAMPs that activate PRRs, resulting in expression of inflammasome components, which causes caspase-1 activation and IL-1β cytokine production through a process mediated by NOD1 or NOD2 and Toll-like receptors (TLRs) [127, 135]. Notably, hypothermia inhibits pyroptosis and thus can alleviate pyroptosis after cerebral ischemia. A151, an inhibitor of cyclic GMP-AMP (cGAMP) synthase (cGAS), markedly reduces the expression of calcitonin gene–related peptides (CGRPs) and decreases the activity of absent in melanoma 2 (AIM2) and the expression of pyroptosis-associated proteins, including GSDMD, caspase-1, IL-1β, and IL-18 [136]. Because pyroptosis is an important neuronal death process, the specific pyrophosphate inhibitor Vx765, which targets the typical inflammasome, has potential therapeutic value for ischemic stroke [137]. These findings show that targeted pyroptosis can improve neurological function in animal models of ischemic stroke. Taken together, these reports suggest that targeting cellular pyroptosis pathways is a novel approach for treating ischemic stroke.

## Role of NLRP3 in COVID-19- or cytokine storm-associated stroke

In November 2019, a severe respiratory illness with a high mortality rate, coronavirus disease 2019 (COVID-19), began to spread worldwide. The pathogen responsible for the disease is severe acute respiratory syndrome coronavirus 2 (SARS-CoV-2). In humans, death from COVID-19 can result from an excessive systemic inflammatory response, or “cytokine storm,” which is a sign of severe disease [138]. In addition, patients with COVID-19 often present with neurological clinical manifestations, including headache, loss of smell, and even stroke. COVID-19 has been suggested to increase the incidence of stroke; the incidence of stroke in critical patients is as high as 6% [139]. In addition, studies have shown that COVID-19-associated ischemic stroke is more severe and has a higher mortality rate than non-COVID-19-associated ischemic stroke [140]. SARS-CoV-2 can affect the nervous system in a number of ways. For example, it can penetrate the CNS through olfactory nerve endings (dysosmia is considered a precursor of COVID-19) [141]. Some authors have suggested that SARS-CoV-2 may enter the lung-gut-brain axis of the vagus nerve or enter the CNS via the hematogenous pathway during viremia in severely affected patients [142, 143]. On the one hand, a large number of inflammatory cytokines are released to destroy the BBB and promote the entry of the virus. Astrocytes can be directly attacked by SARS-CoV-2 [144, 145]. On the other hand, astrocytes and microglia are highly sensitive to systemic proinflammatory cytokines and receive proinflammatory signals from endothelial cells to induce the expression of proinflammatory genes, thereby promoting neuroinflammation and neurodegeneration [142, 146]. The angiotensin-converting enzyme type 2 (ACE-2) receptor has been proven to exist in nerve tissue and vascular endothelial cells; SARS-CoV-2 can bind to brain cells through this receptor and then attack the cells, resulting in nervous system disorders [147]. In human ACE-2-transgenic mice, SARS-CoV-2 can infect neurons and cause neuronal death in an ACE-2-dependent manner [148]. Some studies have also failed to detect SARS-CoV-2 in any cerebrospinal fluid samples, indicating that the nervous system is involved through immune mechanisms rather than through direct viral infection [149]. The levels of various circulating cytokines have been found to be upregulated in the context of COVID-19 through observational studies [150, 151]. In severe cases of SARS-CoV-2 infection, cytokine release syndrome, namely, a cytokine storm, can be observed [139, 152]; the storm is accompanied by increases in circulating C-reactive protein (CRP) levels and erythrocyte sedimentation rates (ESRs) [153]. It is believed that activation of macrophages caused by SARS-CoV-2 infection results in the production and consequent accumulation of inflammatory cytokines, including IFN-α, IFN-γ, IL-6, IL-1β, IL-17, TNF-α, transforming growth factors (TGF-β) and chemokines (CXCL10, CXCL8, CXCL9, CCL2, CCL3, and CCL5). It has also been reported that IL-6 and the NLRP3 inflammasome are the main immune components that mediate the immune response and inflammatory cytokine storm during pathogen infection [154]. We believe that the NLRP3 inflammasome might be one of the triggering factors of the cytokine storm during COVID-19. Viruses, as PAMPs, invade the body and activate dysregulated and excessive immune reactions through a variety of receptors (including NLRs, TLRs and cGAS), which causes overactivation of the NLRP3 inflammasome and then strongly induces the secretion and release of excessive amounts of proinflammatory cytokines and chemokines, resulting in a cytokine storm [155]. The whole SARS-CoV-2 virus or its components can also activate the NLRP3 inflammasome by increasing extracellular ATP levels and activating P2X7R, which is widely expressed in CNS cells, such as microglia and oligodendrocytes. P2X7R activation is induced by increases in angiotensin II (Ang-II) resulting from loss of ACE-2 function due to binding with SARS-CoV-2; the increases in Ang-II lead to activation of the NLRP3 inflammasome, ultimately promoting neuroinvasion and neuroinflammation [156]. Both viruses and their protein components can cross the BBB and enter the CNS to initiate neuroinflammation [157]. Recently, it has been reported that a SARS-CoV-2 protein binds to mannan-binding lectin (MBL) and activates the complement cascade (ComC) and the coagulation cascade (CoaC) through the MBL-MBL-associated serine protease 2 (MASP-2) protease complex, thereby activating the NLRP3 inflammasome [158]. Activated NLRP3 releases DAMPs through GSDMD. For example, high levels of the nuclear protein HMGB1, a DAMP downstream of NLRP3, reflect excessive inflammation during viral infection [159].

COVID-19 is reported to be related to clotting disorders [160]. Coagulation and thrombosis may begin in the lung and other infected organs with endothelial damage, complement activation complicated by the procoagulant effects of IL-6 and neutrophilic granulocyte recruitment [161]. As a result, the levels of D-dimers (fibrinogen breakdown products that form in blood vessels) increase, and disseminated intravascular coagulation occurs [162, 163]. Patients with COVID-19 show hypercoagulability, so SARS-CoV-2 may cause thrombus formation at different sites in patients with thrombotic propensity [164], which may also increase the risk of stroke. In view of the above information, we speculate that targeting the immune cascade, the NLRP3 inflammasome, and hypercoagulability may be beneficial for reducing the incidence of stroke.

## Targeting various components of inflammasome pathways for ischemic stroke treatment

**Fig. 3:**
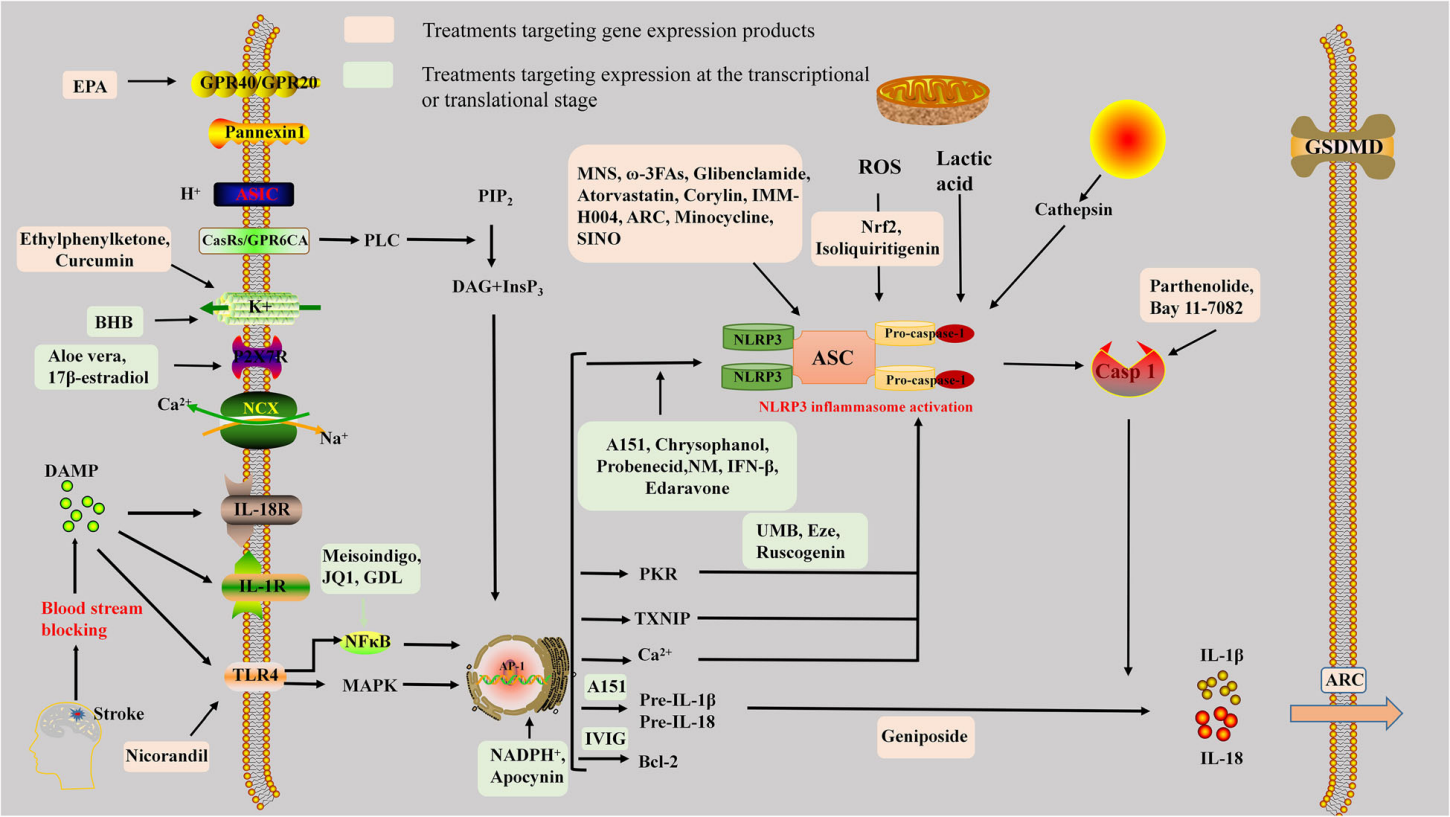
Possible drug actions targeting the different mechanisms of NLRP3 inflammasome activation. EPA: eicosapentaenoic acid; MNS: 3,4-methylenedioxy-beta-nitrostyrene; ω-3FAs: omega-3 fatty acids; ARC: arctigenin; SINO: sinomenine; Nrf2: nuclear factor erythrocyte 2–related factor 2; BHB: β-hydroxybutyrate; NM: nafamostat mesilate; IFN-β: interferon-β; UMB: umbelliferone; Eze: ezetimibe; IVIG: intravenous immunoglobulin; GDL: ginkgo diterpene lactones; DAMP: damage-associated molecular pattern; GPR40/GPR20: G protein–coupled receptor (GPCR) 40/20; ASIC: acid-sensing ion channel; CasR/GPR6CA: Ca^2+^-sensing receptor/GPCR family C group 6 member A; NCX: Na^+^/Ca^2+^ exchanger; IL-18R: interleukin-18 receptor; IL-1R: interleukin-1 receptor; TLR4: Toll-like receptor 4; NF-κB: nuclear factor kappa B; MAPK: mitogen-activated protein kinase; PIP2: phosphatidylinositol-4,5-diphosphate; PLC: phospholipase C; DAG: diacylglycerol; InsP3: inositol triphosphate 3; ROS: reactive oxygen species; ASC: apoptosis-associated speck-like protein with a CARD; PKR: protein kinase R; TXNIP: thioredoxin-interacting protein; Bcl-2: B-cell lymphoma 2; Casp 1: caspase-1; GSDMD: gasdermin D

At present, effective treatments for ischemic stroke are scarce. The current treatment for acute ischemic stroke involves intravenous tPA administration for blood flow restoration. However, this strategy is limited by the narrow therapeutic window and high risk of intracerebral hemorrhage associated with tPA treatment [16]. Oxidative stress and inflammation participate in cerebral IRI, and proper regulation of inflammation could contribute significantly to stroke protection and therapy [13]. Researchers have identified NLRP3 as a critical mediator of neuronal damage and inflammation after stroke, and experiments targeting the NLRP3 inflammasome pathway have shown promising results. These findings have revealed a new path for researchers in the search for effective and reliable treatments for ischemic cerebral apoplexy [165]. The new approaches can be divided into treatments targeting gene products and treatments targeting gene expression (Fig. 3) (Table 1). In addition, studies have demonstrated that distinctive drugs can target different neurocyte types and pathways to yield corresponding effects (Table 2). The feasibility of using small molecules and drugs for stroke therapy needs to be further investigated.

**Table 1:** Inhibitors of the NLRP3 inflammasome in ischemic stroke

Categories	Drugs or molecules
Acting on gene expression products	MCC950, parthenolide, Bay 11-7082, MNS, omega-3 fatty acids, atorvastatin, Nrf2, ethylphenyl ketone, glibenclamide, IMM-H004, eicosapentaenoic acid, geniposide, sinomenine, corylin, minocycline, arctigenin, nicorandil, curcumin
Acting on the process of gene expression	IVIG, aloe vera, A151, chrysophanol, umbelliferone, apocynin, IFN-β, JQ1, meisoindigo, ezetimibe, edaravone, ginkgo diterpene lactones, ketone metabolite hydroxybutyrate, probenecid, nafamostat mesilate, ruscogenin, 17 β-estradiol
Acting on gene expression processes and gene expression products	miR-223, microRNA20a, miR-155-5p, miR-216a, miR-19a-3p, miR-155, miR-874-3p, procyanidins, astragaloside IV, resveratrol, sulforaphane

*MNS*, 3,4-methylenedioxy-beta-nitrostyrene; *Nrf2*, nuclear factor erythrocyte 2–related factor 2; *IVIG*, intravenous immunoglobulin; *IFN-β*, interferon-β

**Table 2:** Cell categories of drugs acting on and related effect/pathways after ischemic stroke

	Categories	Models	Effects/pathways	References
**MCC950**	Microglia	ICH models in mice	Inhibiting ASC oligomerization and secretion and release of Il-1β and IL-18	[166]
**Parthenolide, Bay 11-7082**	Macrophages	Mice BMDM (named NG5)	Inhibiting the activity of caspase-1	[167]
**MNS**	Macrophages	Mice BMDM	Inhibiting ASC speck formation and oligomerization	[168]
**Omega-3 fatty acids**	Macrophages	Mice BMDM	Inhibiting the production ofproinflammatory cytokines	[169]
**Isoliquiritigenin (ILG)**	Neurons	Rats model	Inhibiting NLRP3 inflammasome activation mediated by NF-κB	[78, 170]
**IMM-H004**	Neurons	pMCAO rats model	Anti-inflammatory pathway dependent on CKLF1	[171]
**Eicosapentaenoic acid**	Neurons, Microglia	MCAO mice, OGD model of BV2 cell line	Obstructing GPR40 and GPR20	[172]
**Geniposide**	Microglia	BV2 cell line OGD/R model	Reducing inflammatory cytokine levels; increasing the autophagic activity	[173]
**Sinomenine**	Astrocytes, Microglia	MCAO mice model, Astrocyte, Microglia OGD model	Inhibiting the NLRP3 inflammasome mediated by AMPK pathway	[174]
**Corylin**	Microglia	LPS-induced BV2 cell line inflammation	Alleviating LPS-induced inflammation	[175]
**Minocycline**	Microglia	tMCAO mice model; OGD/R model of BV2 cell line	Stoping microglial activation; inhibiting maturation and release of proinflammatory cytokines	[176]
**Arctigenin**	Neurons	MCAO rats, OGD/R neuron model	Inhibiting sirtuin-1 and decreasing activation of the NLRP3 inflammasome	[177]
**Nicorandil**	Microglia	OGD/R model of BV2 cell line	Opening K^+^-ATP channel; inhibiting the TLR4 signaling pathway	[178]
**Curcumin**	Macrophages	Mice BMDM	Stopping K^+^ efflux; inhibiting caspase-1 activation and IL-1β secretion	[179]
**IVIG**	Neurons, primary cortical neurons	MCAO/R mice; OGD/R model of neuron	Increasing the expression level of the antiapoptotic protein Bcl-2	[24, 180]
**Aloe vera**	Macrophages derived from monocytes		Inhibiting specific signal transduction pathways and proinflammatory cytokines	[181]
**A151**	Macrophage derived from bone marrow	BMDM OGD model	Downregulating the levels of iNOS and NLRP3 inflammasome	[182]
**Chrysophanol**	Neurons	tMCAO mice	Inhibiting the expression of NLRP3, caspase-1, and IL-1β	[183]
**Umbelliferone**	Neurons	MCAO rats	Reducing TXNIP expression	[172]
**Apocynin**	Neurons, Astrocytes, Microglia	MCAO/R model, astrocyte, BV2 cell line	Inhibiting the phosphorylation and degradation of IκBα and nuclear translocation of NF-κBp65	[184]
**IFN-β**	Neurons	tMCAO in IFN-β knockout (IFN-β KO) mice	Inhibiting the STAT1 phosphorylation	[185]
**JQ1**	Neurons, Astrocytes, Microglia	MCAO/R model, astrocyte, BV2 cell line	BRD4 inhibitor; inhibiting NF-κB	[186]
**Meisoindigo**	Neurons, Microglia	MCAO mice stroke model, OGD/R models of HT-22 and BV2 cell lines	Suppressing the TLR4/NF-κB signaling pathway	[187]
**Ezetimibe**	Neurons, Microglia	MCAO rats	Increasing the expression of p-AMPK, Nrf2, and Ho-1 and decreasing that of TXNIP	[188]
**Edaravone**	Neurons, Microglia	Rats ICH	Reducing the generation of IL-1β, caspase-1 and inhibiting NF-κB	[189]
**Ginkgo diterpene lactones**	Neurons, Astrocytes, Microglia	MCAO/R mice, Primary astrocyte OGD/R model	Downregulating of TLR4/NF-κB signaling	[190]
**Ketone metabolite hydroxybutyrate**		BMDM	Inhibiting K^+^ outflow	[191]
**Probenecid**	Astrocytes	Primary astrocyte OGD/R model	Pannexin 1 inhi201caspase-1 and AQP4	[192]
**Nafamostat mesilate**	Microglia	tMCAO rats	Inhibiting the NF-κB signaling pathway and inflammasome activation	[193]
**17 β-estradiol**	Neurons	MCAO/R mice	Reducing the expression of components of the NLC4 inflammasome	[194]
**Procyanidins**	Neurons, Microglia	OGD/R and MCAO/R rats, BV2 cell line	Inhibiting TLR4-NF-κB-NLRP3 signaling pathways	[195]
**Astragaloside IV**	Neurons, Microglia	tMCAO/R mice	Inhibiting TLR4 pathway; reducing ROS production	[196]
**Resveratrol**	Neurons	MCAO/R rats	Downregulating TXNIP expression; decreasing autophagic activity	[84, 197]
**Sulforaphane**	Neurons	MCAO/R rats	Inhibiting NLRP3 inflammasome activity; downregulating the expression of caspase-1, IL-18, and IL-1β	[198]

*MNS*, 3,4-methylenedioxy-beta-nitrostyrene; *IVIG*, intravenous immunoglobulin; *IFN-β*, interferon-β; *pMCAO*, permanent middle cerebral artery occlusion; *tMCAO*, transient middle cerebral artery occlusion; *MCAO/R*, middle cerebral artery occlusion/reperfusion; *OGD/R*, oxygen glucose deprivation/reperfusion; *ICH*, intracerebral hemorrhage; *BMDM*, bone marrow–derived Macrophages; K^+^-ATP channel, *ATP*-sensitive potassium channel; *iNOS*, inducible nitric oxide synthase; *STAT1*, signal transducers and activators of transcription 1; *BRD4*, bromodomain-containing protein 4; *AQP4*, aquaporin 4; *CKLF1*, chemokine-like factor 1; *GPR40*, G protein–coupled receptor 40; *AMPK*, adenosine monophosphate–activated protein kinase; *LPS*, lipopolysaccharide; *TLR4*, Toll-like recepetor; *IL-1β*, interleukin-1β; *TXNIP*, thioredoxin-interaction protein; *NF-κB*, nuclear factor kappa B; *ROS*, reactive oxygen species

### Treatments targeting gene expression products to impact NLRP3 inflammasome activation

MCC950, which is known for its action on the NLRP3 inflammasome, can inhibit not only the ASC oligomerization induced by typical or atypical NLRP3 activation but also the processing, secretion, and release of Il-1β and IL-18 [166]. During ischemic stroke, MCC950 can reduce neurological defects and cerebral edema, improve the integrity of the BBB, and decrease neuronal and glial cell death after stroke [199]. In addition, the NLRP3 receptor can be excited by K^+^ efflux; thus, suppressing K^+^ efflux or increasing the extracellular K^+^ concentration can inhibit NLRP3 receptor activation. Research has shown that suppression of voltage-gated K^+^ channels (using ethyl phenyl ketone) prevents activation of the NLRP3 receptor in mouse macrophages [42, 200]. In addition, a study has reported that glibenclamide inhibits the NLRP3 inflammasome and effectively reduces edema development [201]. Moreover, nicorandil, an ATP-sensitive K^+^ channel (K^+^ ATP) opener, inhibits the TLR4 signaling pathway and NLRP3 inflammasome activation, thereby reducing IL-1β production. In in vitro experiments, nicorandil has been shown to inhibit inflammasome activation and TLR4 signal transduction to combat oxygen-glucose deprivation (OGD)-induced neuroinflammation [178]. However, the roles of various K^+^ channels in the treatment of stroke still need to be explored in more detail. 3,4-Methylenedioxy-β-nitrostyrene (MNS) has been shown to specifically stop NLRP3-induced ASC speck formation and oligomerization, but it does not block the K^+^ flow induced by NLRP3 agonism. Moreover, NLRP3 ATPase activity can be inhibited by the administration of MNS in vitro, indicating that MNS blocks the NLRP3 inflammasome to alleviate the inflammatory response [168]. Parthenolide and Bay 11-7082 directly inhibit activation of the protease caspase-1 to inhibit the various inflammasomes in macrophages [167]. Omega-3 fatty acids also arrest the NLRP3 inflammasome-dependent inflammatory response [169]. A randomized controlled study has shown that immediate administration of atorvastatin after atherosclerotic ischemic stroke significantly reduces the activation of immune inflammation in the acute phase of stroke [202]. The results of an experimental study have suggested that geniposide might reduce inflammatory cytokine levels and inhibit NLRP3 inflammasome activation by increasing the autophagic activity of the BV2 microglial cell line, thus reducing the inflammatory response after stroke [173]. Corylin inhibits NLRP3 inflammasome activation and alleviates lipopolysaccharide (LPS)-induced inflammation in LPS-activated BV2 cells [175]. Curcumin, a common suppressor of NLRP3 inflammasome activation, inhibits NLRP3 by stopping K^+^ efflux and interfering with downstream events, which in turn inhibits caspase-1 activation and IL-1β secretion [179]. Some drugs can indirectly inhibit NLRP3 inflammasome activation through molecular mediation and thus inhibit downstream inflammatory cytokines. In a standard rat model of cerebral ischemia, IMM-H004 (a novel coumarin derivative) has been found to provide significant protection against cerebral ischemia via an anti-inflammatory pathway dependent on chemokine-like factor 1 (CKLF1). IMM-H004 downregulates the binding of CKLF1 to C-C chemokine receptor type 4, further inhibiting NLRP3 inflammasome activation and the subsequent inflammatory response and ultimately protecting against ischemic damage. This evidence provides support for human clinical trials studying IMM-H004 for the treatment of acute cerebral ischemia [171]. Arctigenin (ARC) therapy effectively inhibits the activation of the NLRP3 inflammasome and prevents the secretion of IL-1β and IL-18 caused by ischemic stroke in vivo and in vitro. The effects of administration of a specific silent information regulator 1 (sirtuin-1/sirt-1) inhibitor, EX527, prove that ARC protects against cerebral ischemia by inhibiting activation of the NLRP3 inflammasome in a sirtuin-1-dependent manner [177]. After 1 h of ischemia-reperfusion, minocycline can stop microglial activation and weaken damage caused by middle cerebral artery occlusion (MCAO) by inhibiting NLRP3 inflammasome activation and proinflammatory cytokine maturation/release, ameliorating neurological disorders, reducing infarct volumes, decreasing cerebral edema, and relieving ischemic brain injury [176]. Sinomenine (SINO) inhibits the NLRP3 inflammasome via the adenosine monophosphate-activated protein kinase (AMPK) pathway, thus exerting a neuroprotective effect in ischemic stroke by alleviating cerebral infarction, cerebral edema, neuronal apoptosis, and neurological impairment after MCAO [174]. NRF2 functions at the initiation step to downregulate ROS-induced activation of the NLRP3 inflammasome. Isoliquiritigenin inhibits NLRP3 inflammasome activation mediated by ROS and NF-κB by promoting the antioxidant system of NRF2 and alleviates early brain damage after hemorrhage [78, 170]. Eicosapentaenoic acid (EPA) suppresses NLRP3 inflammasome activation by obstructing G protein–coupled receptor 40 (GPR40) and G protein–coupled receptor 120 (GPR120); importantly, EPA can ameliorate apoptosis induced by acute cerebral infarction [203].

### Treatments targeting expression at the transcriptional or translational stage

In addition to treatments targeting gene expression products, there are also some small molecules or drugs that act on gene expression processes to regulate inflammatory responses by upregulating or downregulating the expression of various downstream proteins. In ischemic stroke models, treatment with intravenous immunoglobulin (IVIG) has been found to reduce neuronal cell death and infarct volume and to ameliorate brain function. In these models, IVIG increases the expression level of the antiapoptotic protein BCL-2, which inhibits the NLRP3 receptor by blocking binding between ATP and the NACHT domain in the NLRP3 receptor, downregulates proinflammatory cytokine expression, and protects neurons and cerebral tissue [24, 180]. A151 (a synthetic oligodeoxynucleotide containing multiple distal TTAGGG sequences) decreases maturation of IL-1β and caspase-1, reduces the generation of IL-1β, downregulates the levels of inducible nitric oxide synthase (iNOS) and NLRP3, and inhibits depolarization of the intracellular mitochondrial membrane potential in activated myeloid-derived macrophages stimulated with OGD and LPS [182]. In the ischemic cerebral cortex, NADPH and apocynin inhibit phosphorylation and degradation of IκBα; nuclear translocation of NF-κBp65; expression of NF-κBp65 targets, such as iNOS and cyclooxygenase (COX2); and expression of inflammasome proteins, including NLRP3, ASC, and caspase-1. These effects have been found to greatly decrease infarct size in a mouse stroke model, increase survival after stroke, restore neurological function, and provide relatively strong neuroprotective effects during ischemic stroke [184]. Chrysophanol, an extract of rhubarb, has a variety of pharmacological effects, including anti-inflammatory effects. In a transient MCAO (tMCAO) mouse model, chrysophanol has been shown to inhibit the expression of NLRP3, caspase-1, and IL-1β and to protect against ischemic brain injury [183]. Umbelliferone (UMB) therapy reduces TXNIP expression, inhibits NLRP3 inflammasome activation, and has a beneficial neuroprotective effect against focal cerebral ischemia [172]. Ezetimibe (Eze) increases the expression of p-AMPK, NRF2, and Ho-1 and decreases that of TXNIP, NLRP3, caspase-1, and IL-1β, thus inhibiting oxidative stress and subsequent neuroinflammation to protect brain tissues. In addition, ruscogenin inhibits IL-1β, NLRP3, caspase-1, and TXNIP expression; reduces ROS production; blocks MAPK pathway activity; relieves edema in the cerebral obstruction area; improves impairments in neurological function; increases cerebral blood flow (CBF); alleviates histopathological injury; and upregulates tight junction component expression. These findings provide a new perspective for the therapy of ischemic stroke [188]. Meisoindigo, a derivative of indirubin, significantly inhibits NLRP3 inflammasome activation and blocks the M1 polarization of microglia/macrophages by suppressing the TLR4/NF-κB signaling pathway in a dose-dependent manner, thus reducing ischemic brain injury caused by stroke [187]. Ginkgo diterpene lactones (GDLs) inhibit platelet aggregation, astrocyte activation, and proinflammatory cytokine release, which may be positively related to the downregulation of TLR4/NF-κB signaling [190]. The channel pannexin 1 can activate inflammasomes in astrocytes and participate in the process of ischemic injury. Probenecid, a pannexin 1 inhibitor, reduces the expression levels of NLRP3, caspase-1, and aquaporin 4 (AQP4) in OGD astrocyte models and inhibits IL-1β release; probenecid contributes to a neuroprotective effect against ischemic damage by inhibiting inflammasome activity and reducing astrocyte edema [192]. Nafamostat mesilate (NM) is a broad-spectrum serine protease inhibitor. Administration of NM leads to inhibition of proinflammatory mediators and promotion of anti-inflammatory mediators; these effects may be partly attributable to the immunoregulatory effects of NM, which involve NF-κB signaling pathway inhibition and inflammasome activation [193]. Aloe vera (an immunomodulator) dose-dependently inhibits the production of pro-IL-1β, caspase-1, NLRP3, and P2X7R by inhibiting specific signal transduction pathways and reduces the release of IL-8, TNF-α, IL-6, and IL-1β in primary macrophages induced by LPS [181]. 17-Estradiol remarkably reduces the expression of components of the NLRC4 inflammasome, AIM2, ASC, NLRP3, IL-1β, IL-18, TNF-α, and P2X7R at the gene and protein levels after global cerebral ischemia (GCI) [194]. Limiting inflammation after stroke has been documented to reduce neuronal death or improve nerve function, and IFN-β has been recommended as a candidate for stroke treatment. As IFN-β signaling weakens inflammation and mediates peripheral immune cell activity, it may positively affect stroke outcomes [185]. Studies have suggested that IFN-β may inhibit IL-1β production partly by inhibiting phosphorylation of the transcription factor signal transducer and activator of transcription 1 (STAT1), thereby inhibiting the NLRP3 inflammasome and caspase-1-dependent IL-1β maturation; thus, IFN-β targeting might be a new approach to alleviate ischemic injury [204]. JQ1, a bromodomain-containing protein 4 (BRD4) inhibitor, can reduce the generation of proinflammatory agents by inhibiting NF-κB, pyroptosis, and inflammasome activation, as shown by significantly reduced NLRP3, ASC, caspase-1, and GSDMD levels in MCAO mice; these effects can reduce infarct volumes, brain water content and neural function defects in MCAO mice and protect against brain injury induced by cerebral ischemia [186]. Moreover, edaravone reduces NLRP3 expression in microglia. Additionally, edaravone has neuroprotective effects similar to those of MCC950 and reduces the generation of IL-1β, caspase-1, and NF-κB at the protein or gene level, significantly alleviating cerebral edema and improving neurological deficits in rats after cerebral hemorrhage [189]. Another molecule, the ketone metabolite β-hydroxybutyrate (BHB), exerts an anti-inflammatory effect by inhibiting K^+^ outflow from macrophages and reducing ASC oligomerization and speck formation, thereby inhibiting the generation of IL-1β and IL-18 in human mononuclear cells induced by NLRP3-induced ASC oligomers [191].

### Treatments targeting gene expression processes and products

miR-223 inhibits activation of the NLRP3 inflammasome and generation of IL-1β by binding to three conserved sites in the untranslated region (UTR), thereby inhibiting protein expression [205–207], and downregulation of caspase-1 and IL-1β expression reduces brain edema and improves nerve function [208]. miR-155-5p targets DUSP14 to promote cerebral IRI by regulating the NF-κB and MAPK signaling pathways, and inhibition of miR-155-5p is significantly effective in treating injured brain tissue. Therefore, research suggests that miR-155-5p may be a new target for ischemic stroke therapy [209]. Overexpression of miR-216a inhibits Janus tyrosine kinase 2 (JAK2) protein levels in OGD/reoxygenation (OGD/R)-subjected neurons and ischemic areas in MCAO models, negatively regulates JAK and JAK2/STAT3 pathways, reduces ischemic infarction incidence, and alleviates neurofunctional impairment [210]. miR-19a-3p promotes I/R-induced inflammation and apoptosis by targeting insulin-like growth factor binding protein 3 (IGFBP3), as demonstrated by the finding that miR-19a-3p inhibitors exert protective effects against cerebral IRI by inhibiting apoptosis and reducing inflammation, ultimately reducing infarct volume and improving nerve function and activity [211]. Knockdown of miR-155 expression reduces the severity of IRI by inhibiting the inflammatory response and improving neurological function, and miR-155 downregulation reduces OGD-induced injury by increasing proliferation, inhibiting apoptosis, and inhibiting inflammatory factor (TNF-α, IL-1β, IL-6, iNOS, and COX-2) expression [212, 213]. miR-874-3p negatively regulates BCL-2-modifying factor (BMF) and BCL-2 family proteins to reduce the severity of ischemic injury [214]. Other miRNAs, including miRNA-133a-1 and miRNA-377, are also involved in NLRP3 inflammasome activation [215, 216]. In summary, different miRNAs have different effects on NLRP3 inflammasome activation and stroke outcome. Reducing miR-155-5p, miR-19a-3p, miR-155, miRNA-133a-1 and miRNA-377 levels may be of great value in treating inflammation associated with ischemic stroke. In contrast to these miRNAs, miR-223, miR-216a, and miR-874-3p suppress NLRP3 inflammasome activation and have beneficial effects. Procyanidins significantly inhibit in vivo and in vitro activation of MCAO/reperfusion (MCAO/R)- and OGD/R-mediated TLR4-NF-κB-NLRP3 signaling pathways; inhibit IL-1β production; significantly improve neurological deficits; and reduce cerebral edema, cerebral infarct size, and apoptosis [195]. In addition to miRNAs, a few compounds can also act on both gene expression processes and gene expression products related to NLRP3 inflammasome activation to influence ischemic brain injury. For example, astragaloside IV reduces the expression of TLR4 and its downstream adaptor proteins, including MyD88, Toll/IL-1β receptor domain-containing adaptor-inducing interferon-β (TRIF), and tumor necrosis factor receptor-associated factor 6 (TRAF6), thereby inhibiting NF-κB phosphorylation, reducing ROS production and in turn inhibiting NLRP3 activation [196]. Resveratrol downregulates TXNIP expression; reduces poly-ADP-ribose polymerase (PARP) activity; decreases autophagic activity; and inhibits NLRP3 activation, caspase-1 activation, and IL-1β release to significantly reduce cerebral infarct volume [84, 197]. Moreover, sulforaphane inhibits NLRP3 inflammasome activation and downregulates caspase-1, IL-18, and IL-1β expression, which improves prognosis after focal cerebral ischemia and consequently alleviates brain injury [198].

### Treatments involving stem cells and biological products

After cerebral ischemia occurs, immune system activation, gene expression profile alteration, BBB destruction, immune cell infiltration, and cytokine production also occur. These events indicate that immune cells and even stem cells are important in ischemic stroke. Application of stem cells to control the immune-inflammatory response is a new treatment approach for ischemic stroke. Human cord blood–derived multipotent stem cells (HCB-SCs) cocultured with lymphocytes promote regulatory T cell (Treg) differentiation. Cocultured cell transplantation reduces the expression of NLRP3 and related factors, blocks activation of the NLRP3 inflammasome in neurons, suppresses the activity of NF-κB and extracellular signal-regulated kinase (ERK) in ischemic brain tissues, significantly improves nerve function defects, and reduces ischemic brain damage, indicating that this approach may be a promising treatment strategy for ischemic stroke [217]. Moreover, mesenchymal stem cell (MSC) therapy significantly reduces JNK phosphorylation induced by ischemia, showing that this treatment has antiapoptotic and anti-inflammatory effects [218]. In addition, experiments have shown that Tregs may have a neuroprotective role in ischemic stroke by inhibiting inflammation and effector T cell activation [219]. IL-33 can activate Tregs, which produces a neuroprotective effect related to reductions in apoptosis-related protein expression as well as generation and activation of St2-dependent Tregs and Treg-related cytokines [220]. Research has shown that autologous bone marrow MSCs have lower immunogenicity and produce weaker immune responses than allogeneic bone marrow MSCs and can better promote recovery and reduce infarct volume in MCAO rats [221]. Nonhematopoietic umbilical blood stem cells (nh-UCBSCs) have also been demonstrated to protect ischemic brain tissues by inhibiting immune cells from undergoing peripheral migration into the brain or by downregulating abnormal activation of immune responses [222]. nh-UCBSCs have shown enormous potential in stroke treatment and exhibit an enhanced therapeutic window; these cells also have no known side effects and can be stored, and their production can be scaled for extensive use in stroke treatment [223].

### Other treatments

In one study, MTS510 (an anti-TLR4 antibody) was administered through different routes in vivo to study MCAO in adult male wild-type mice (tMCAO range: 45 minutes to 2 days); the results showed that intravascular administration of MTS510 to mice subjected to 45 minutes of MCAO increased neurological function, decreased infarct volume, and reduced brain swelling, suggesting that blocking TLR4 by using specific monoclonal antibodies is a promising stroke treatment strategy [224]. In MCAO mouse models, activation of GPR120 protects against focal ischemic brain injury by preventing inflammation and apoptosis [225]. In vivo results have shown that notoginseng leaf triterpene (PNGL) pretreatment significantly reduces infarct size, decreases brain water content, and improves nerve function in MCAO/R model rats. In addition, PNGL pretreatment significantly reduces BBB damage; inhibits neuronal apoptosis and neuronal loss caused by cerebral IRI; and significantly reduces the serum concentrations of IL-6, TNF-α, IL-1β, and HMGB1 in a dose-dependent manner. These findings suggest that inhibition of inflammation, which may be associated with MAPK inhibition and NF-κB activation, may be involved in the neuroprotective effect of PNGL [226]. In a rat model of MCAO, hispidulin has been found to improve neurological symptoms after cerebral IRI, thereby reducing the infarct area and cerebral edema. Hispidulin plays a neuroprotective role by modulating AMPK/glycogen synthase kinase (GSK) 3 signaling to inhibit NLRP3-mediated pyroptosis in vitro and in vivo [227].

## Expectations

The brain is a complex organ with various components that engage in crosstalk to form a network that affects the brain itself. The NLRP3 inflammasome is responsible for exerting adverse effects on neurons after ischemic stroke. In addition to inflammasomes, ERS, autophagy, ferroptosis, oxidative stress, and other excessive physiological and pathological processes that are closely related to the NLRP3 inflammasome cause neuronal death. The ER is an organelle that regulates protein folding homeostasis by folding and modifying secretory and membrane proteins [228]. Exposure of cells to various stress signals disrupts ER homeostasis and causes dysfunction. Upon reception of stress signals, the ER triggers a protective or adaptive response known as the unfolded-protein response (UPR) in order to recover ER stability. However, severe ERS induces mitochondrial Ca^2+^ overload, ROS accumulation, and ATP depletion, thereby activating mitochondrial-dependent apoptosis [229, 230]. In addition, misfolded/abnormal proteins in cells trigger the UPR pathway, which may result in severe loss of neuronal function and viability [231]. ERS and oxidative stress jointly lead to activation of the NLRP3 inflammasome in neurons, causing inflammatory responses [232]. Inhibiting NLRP3 activation induced by ERS can protect neurons from ischemic injury and thus exert a neuroprotective effect after stroke [233, 234]. However, NLRP3 is involved in ERS-induced mitochondrial damage; thus, the NLRP3 inflammasome can also induce ERS, forming a feedback loop that further promotes inflammation [235, 236].

Autophagy is a physiological destruction process that differs from necrosis and apoptosis. The main characteristic of autophagy is the formation of autophagosomes. Upon encountering a series of stress conditions, cells attempt to maintain a stable intracellular environment and normal cell function by degrading cytoplasmic components [237, 238]. Stroke results in the production and activation of many stress factors, including ROS, as well as misfolding and abnormal accumulation of proteins. These factors induce autophagy [239, 240]. In addition, ERS can directly activate autophagy. Through autophagy, abnormal proteins are destroyed in lysosomal-dependent pathways to restore homeostasis [241]. Activated autophagy negatively regulates activation of the NLRP3 inflammasome. Conversely, autophagy dysfunction can lead to activation of the NLRP3 inflammasome, and inhibiting GSK-3β to enhance autophagy can inhibit NLRP3 inflammasome activation and reduce IRI [242, 243]. Autophagy function is impaired after ischemic brain injury, but progesterone and geniposide can inhibit the activation and expression of the NLRP3 inflammasome and increase autophagic activity [173, 243]. These results indicate that autophagy can negatively regulate the activation of the NLRP3 inflammasome. However, autophagy is a double-edged sword; it can not only play a neuroprotective role but also induce the NLRP3 inflammasome cascade through its overactivation resulting from excessive ER initiation, excessive ROS production and subsequent NLRP3 inflammasome activation [244]. Inhibition of autophagy or knockout of autophagy genes, such as LC3, results in inactivation of the NLRP3 inflammasome [245]. Autophagy regulates the activation of the NLRP3 inflammasome, and vice versa [246, 247]. For example, on the one hand, the occurrence of autophagy is dependent on the NLRP3 inflammasome sensor, so the NLRP3 inflammasome can activate autophagy [248]. On the other hand, the NLRP3 inflammasome commonly inhibits activation and diminishes the neuroprotective effect of autophagy through mature caspase-1-mediated cleavage of TRIF, a vital molecule in the TLR4-TRIF signaling pathway, which mediates autophagy activation [247, 249, 250].

Ferroptosis is a newly discovered form of Fe^2+^-dependent cell death that can lead to programmed neuronal death. Ferroptosis is induced by cellular redox imbalance, inhibition of glutathione peroxidase 4 (GPX 4) activity, and ultimate accumulation of lipid peroxides, which induces damage to cell structure and function and leads to cell death [251–253].

**Fig. 4:**
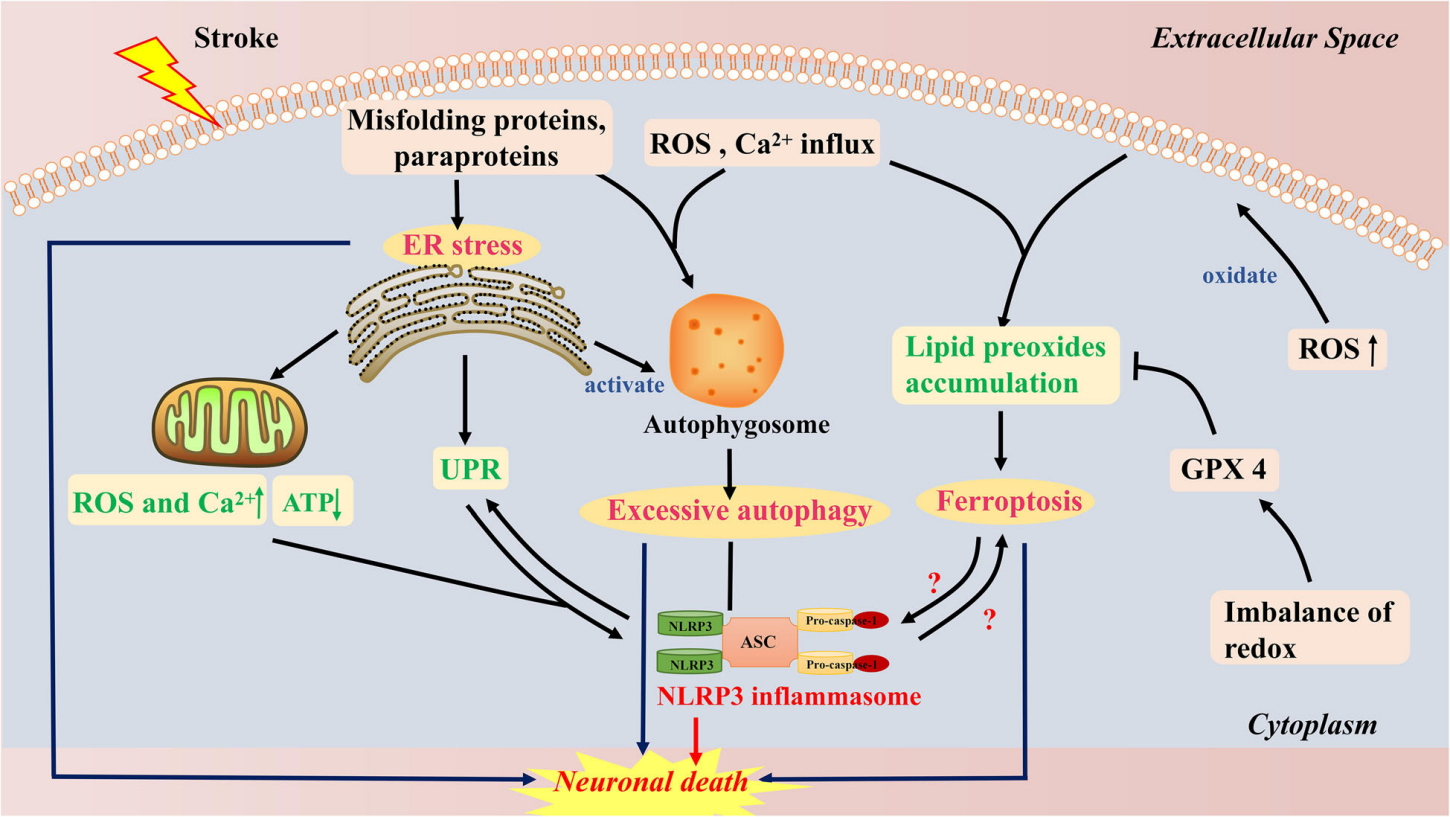
Crosstalk among several physiological and pathological processes leads to neuronal death after stroke. Misfolded proteins and paraproteins trigger ER stress and the UPR to activate the NLRP3 inflammasome and aggravate inflammatory responses; the NLRP3 inflammasome can also promote the UPR and ER stress. ROS accumulation, Ca^2+^ dyshomeostasis, and ER stress excessively activate autophagy. Autophagy normally inhibits the NLRP3 inflammasome but can induce NLRP3 inflammasome activation when it is excessive. The NLRP3 inflammasome can also act on autophagy. Lipid peroxide accumulation results in ferroptosis, and there is probably crosstalk between ferroptosis and NLRP3 inflammasome activation. ER stress, excessive autophagy, ferroptosis, and the NLRP3 inflammasome together form an LNAS

ERS and autophagy, through NLRP3 inflammation, influence the death of neurons after stroke and together act on cells to produce comprehensive and complex effects. Whether ferroptosis causes neuronal death through the NLRP3 inflammasome is unclear. Autophagy can induce ferroptosis by interfering with cellular iron homeostasis and by enhancing lipid peroxide and ROS generation, and autophagy simultaneously activates the NLRP3 inflammasome. Does ferroptosis induce NLRP3 inflammasome activation, or do these processes interact? Ferroptosis is a kind of iron accumulation- and ROS-dependent cell death, and ROS production is a nonnegligible mechanism of NLRP3 inflammasome activation. NLRP3 inflammasome activation is accompanied by ferroptosis [254], and antioxidants or inhibitors of ferroptosis can inhibit the NLRP3 inflammasome [255]. It has also been documented that myrrh exerts a neuroprotective effect by regulating the TXNIP/NLRP3 axis in ischemic stroke to reduce ROS-mediated ferroptosis [256]. Therefore, we hypothesize that activation of the NLRP3 inflammasome may be related to ferroptosis and that these processes may engage in crosstalk. However, more research is needed to test this hypothesis. We propose that various effects in the body interact with each other to form a lethal network of neurons after stroke (LNAS) (Fig. 4). It is possible that no drugs with satisfactory clinical effects have been found so far because the existing drugs act only on a single site, neglecting the complexity of the physiological regulatory network and the neuronal death network after stroke. This possibility emphasizes that each organism should be considered as a whole, that various factors should be comprehensively assessed, and that more attention should be paid to the relationships among different aspects in future research.

## Conclusion

Recent studies have identified NLRP3 inflammasome-mediated ischemic stroke as a new mechanism that leads to neuron and glial cell death after brain injury. This inflammatory mechanism, which involves the innate immune system, not only causes brain tissue damage but also plays a beneficial role in brain tissue recovery. After cerebral vascular obstruction or thromboembolism, cerebral tissue is damaged due to ischemia and hypoxia, and cell stress reactions, including intracellular K^+^ efflux, mitochondrial injury, high ROS production, lysosomal rupture, and increases in intracellular Ca^2+^ levels, occur. Through various channel components (such as P2X7R and TXNIP) and signaling pathways (the NF-κB and MAPK pathways), these changes activate NLRP3, leading to NLRP3-mediated cleavage and production of caspase-1 from pro-caspase-1, which is followed by processing of pro-IL-1β and pro-IL-18 into mature inflammatory cytokines. Various components are involved in stress responses and the inflammatory process, and many drugs and molecules that inhibit the NLRP3 inflammasome and reduce the inflammatory response in the context of ischemic brain injury have been identified. The effectiveness of these agents has been demonstrated in several experiments, as described above. The brain is a complex organ, and its homeostasis is maintained by a complicated network of multifarious physiological and pathological mechanisms. Understanding the crosstalk of the NLRP3 inflammasome with other entities or mechanisms may be beneficial for the development of effective therapeutic strategies for ischemic stroke.

## Data Availability

Not applicable.

## Abbreviations

ACE-2: Angiotensin-converting enzyme type 2
AIM2: Absent in melanoma 2
AMPK: Adenosine monophosphate-activated protein kinase
Ang-II: Angiotensin II
AQP4: Aquaporin 4
ARC: Arctigenin
ASC: Apoptosis-associated speck-like protein with a CARD
ASIC: Acid-sensitive ion channel
ATP: Adenosine triphosphate
BBB: Blood-brain barrier
BBG: Brilliant Blue G
BHB: β-Hydroxybutyrate
BMDM: Bone marrow–derived macrophage
BMF: BCL-2-modifying factor
BMI: Body mass index
BRD4: Bromodomain-containing protein 4
C5aR2: Complement 5a receptor 2
cAMP: Cyclic adenosine monophosphate
CaSR: Ca^2+^-sensing receptor
CBF: Cerebral blood flow
cGAMP: Cyclic GMP-AMP
cGAS: cGAMP synthase
CGRP: Calcitonin gene–related peptide
CKLF1: Chemokine-like factor 1
CNS: Central nervous system
CoaC: Coagulation cascade
ComC: Complement cascade
COX2: Cyclooxygenase 2
CRP: C-reactive protein
Cyt: Cytochrome-c
DAG: Diacylglycerol
DAMP: Damage-associated molecular pattern
dsRNA: Double-stranded RNA
EPA: Eicosapentaenoic acid
ER: Endoplasmic reticulum
ERK: Extracellular signal-regulated kinase
ESR: Erythrocyte sedimentation rate
Eze: Ezetimibe
GCI: Global cerebral ischemia
GDL: Ginkgo diterpene lactone
GPCR: G protein–coupled receptor
GPR120: GPCR 120
GPR40: GPCR 40
GPR6CA: GPCR family C group 6 member A
GSDMD: Gasdermin D
HCB-SC: Human cord blood–derived multipotent stem cell
HMGB1: High-mobility group box 1
I/R: Ischemia/reperfusion
IGFBP3: Insulin-like growth factor–binding protein 3
IL: Interleukin
iNOS: Inducible NOS
InsP3: Inositol triphosphate
IRI: I/R injury
IVIG: Intravenous immunoglobulin
JAK2: Janus tyrosine kinase 2
LDL-C: Low-density lipoprotein cholesterol
LNAS: Lethal network of neurons after stroke
LPS: Lipopolysaccharide
LRR: Leucine-rich repeat
MAC: Membrane attack complex
MAPK: Mitogen-activated protein kinase
MBL: Mannan-binding lectin
MCAO: Middle cerebral artery occlusion
MCU: Mitochondrial calcium uniporter
MNS: 3,4-Methylenedioxy-β-nitrostyrene
MSC: Mesenchymal stem cell
NACHT: NAIP, CIITA, HET-E and TP1
NADPH: Nicotinamide adenine dinucleotide phosphate
NCX: Na^+^/Ca^2+^ exchanger
NF-κB: Nuclear factor kappa B
nh-UCBSC: Nonhematopoietic umbilical blood stem cell
NLR: NOD-like receptor
NLRP3: NLR family pyrin domain-containing 3
NM: Nafamostat mesilate
NMDAR: N-Methyl-D-aspartate receptor
NOD: Nucleotide-binding oligomerization domain
NOS: Nitric oxide synthase
NOX: NADPH oxidase
NRF2: Nuclear factor erythrocyte 2–related factor 2
OGD: Oxygen-glucose deprivation
P2X7R: Purinergic ligand-gated ion channel 7 receptor
PA: Palmitic acid
PAMP: Pathogen-associated molecular pattern
PARP: Poly-ADP-ribose polymerase
PIP2: Phosphatidylinositol-4,5-diphosphate
PKR: Protein kinase R
PLA2: Phospholipase A2
PLC: Phospholipase C
PNGL: Notoginseng leaf triterpene
PRR: Pattern recognition receptor
PYD: N-Terminal pyrin domain
REC: Retinal endothelial cell
RIP1: Receptor-interacting protein 1
ROS: Reactive oxygen species
sc-tPA: Single-chain form of tPA
SINO: Sinomenine
Sirtuin-1: Silent information regulator 1
SOCE: Store-operated Ca^2+^ entry
STAT1: Signal transducer and activator of transcription 1
TLR: Toll-like receptor
TLR4: Toll-like receptor 4
tMCAO: Transient MCAO
tPA: Tissue plasminogen activator
TRAF6: Tumor necrosis factor receptor-associated factor 6
TRIF: Toll/IL-1β receptor domain-containing adaptor-inducing interferon-β
TRX: Thioredoxin
TXNIP: TRX-interacting protein
UMB: Umbelliferone
UTR: Untranslated region
